# Author Correction: Universal toxin-based selection for precise genome engineering in human cells

**DOI:** 10.1038/s41467-021-23345-z

**Published:** 2021-05-10

**Authors:** Songyuan Li, Nina Akrap, Silvia Cerboni, Michelle J. Porritt, Sandra Wimberger, Anders Lundin, Carl Möller, Mike Firth, Euan Gordon, Bojana Lazovic, Aleksandra Sieńska, Luna Simona Pane, Matthew A. Coelho, Giovanni Ciotta, Giovanni Pellegrini, Marcella Sini, Xiufeng Xu, Suman Mitra, Mohammad Bohlooly-Y, Benjamin J. M. Taylor, Grzegorz Sienski, Marcello Maresca

**Affiliations:** 1 Translational Genomics, Discovery Sciences, BioPharmaceuticals R&D, AstraZeneca, Gothenburg, Sweden; 2 Translational Science and Experimental Medicine, Respiratory & Immunology, BioPharmaceuticals R&D, AstraZeneca, Gothenburg, Sweden; 3 Department of Chemistry & Molecular Biology, University of Gothenburg, Gothenburg, Sweden; 4 R&D Data Infrastructure & Tools, AstraZeneca, Cambridge, UK; 5 Discovery Biology SWE, Discovery Sciences, BioPharmaceuticals R&D, AstraZeneca, Gothenburg, Sweden; 6 Oulu Center for Cell‐Matrix Research, Biocenter Oulu and Faculty of Biochemistry and Molecular Medicine, University of Oulu, Oulu, Finland; 7 Wellcome Sanger Institute, Cambridge, UK; 8 Discovery Biology UK, Discovery Sciences, BioPharmaceuticals R&D, AstraZeneca, Cambridge, UK; 9 CVRM pathology, Clinical Pharmacology & Safety Sciences, BioPharmaceuticals R&D, AstraZeneca, Gothenburg, Sweden; 10 Department of Biosciences and Nutrition, Karolinska Institute, Stockholm, Sweden; 11 Inserm UMR1277 CNRS UMR9020 – CANTHER, Institut pour la Recherche sur le Cancer de Lille, Lille, France

**Keywords:** Biological techniques, Gene delivery, Genetic engineering, Genetic techniques, Biotechnology

Correction to: *Nature Communications* 10.1038/s41467-020-20810-z, published online 21 January 2021.

In the original version of this Article the Acknowledgements failed to state support from the European Union’s Horizon 2020 research and innovation program by grant number 814316. This has been fixed the HTML and PDF versions of the Article.

