# Supplementary material for: Universal toxin-based selection for precise genome engineering in human cells
Source: Nat Commun. 2021 Jan 21;12:497. doi: 10.1038/s41467-020-20810-z (PMC7820243; doi:10.1038/s41467-020-20810-z)
Supplement: Supplementary file 1 — Supplementary Figures and Tables [file 41467_2020_20810_MOESM1_ESM.pdf]

Universal toxin-based selection for precise genome engineering in human cells

Li et al

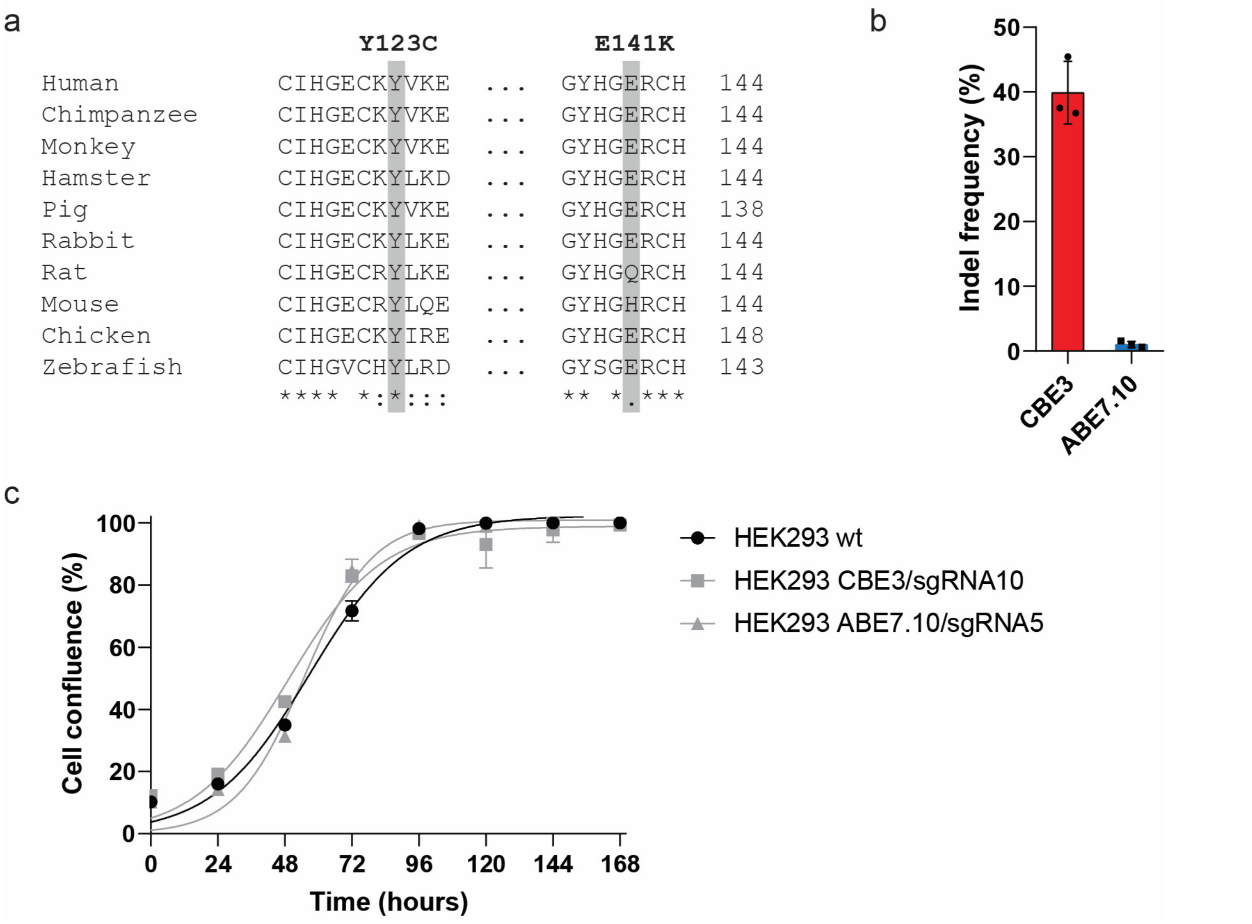

Supplementary Figure 1. **Identification and characterization of *HBEGF* resistant mutations.** (a). Alignment of HBEGF homologs from different species. (b) Indel frequencies observed in DT-resistant populations generated with CBE3/sgRNA10 pair or ABE7.10/sgRNA5 pair. (c) Cell proliferation curves of HEK293 wildtype cells (HEK293 wt) and DT-resistant cells generated by CBE3/sgRNA10 (HEK293 CBE3/sgRNA10), and ABE7.10/sgRNA5 (HEK293 ABE7.10/sgRNA5), respectively. Cell confluence was measured in 96-well plates and quantified using the IncuCyte S3 live cell analysis system (Essen BioScience).

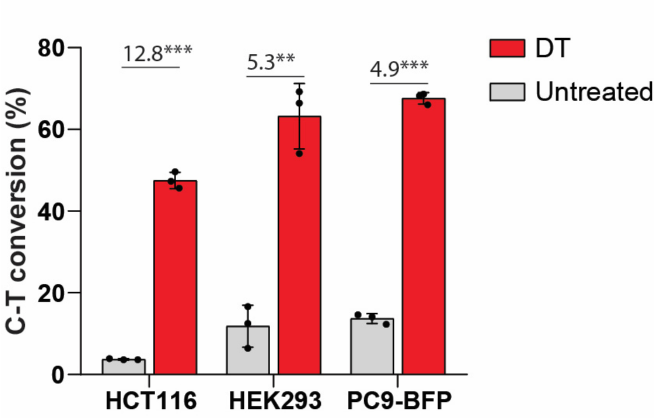

Supplementary Figure 2. **CBE co-selection in different cell lines.** CBE3/sgRNA targeting *PCSK9*, CBE3/sgRNA targeting *PCSK9*, CBE3/sgRNA targeting BFP were transfected into HCT116, HEK293 and PC9-BFP cells, respectively. Genomic DNA was extracted from cells selected or unselected with DT (20 ng/mL) and analyzed by Amplicon-Seq. Values and error bars reflect mean ± s.d. of n=3 independent biological replicates. Relative fold-changes are indicated in the graphs. *P< 0.05, **P< 0.01, ***P<0.001, Student's paired t-test (two-tailed). P values are calculated as below: HCT116 (0.0006), HEK293 (0.0028), PC9-BFP (0.0007).

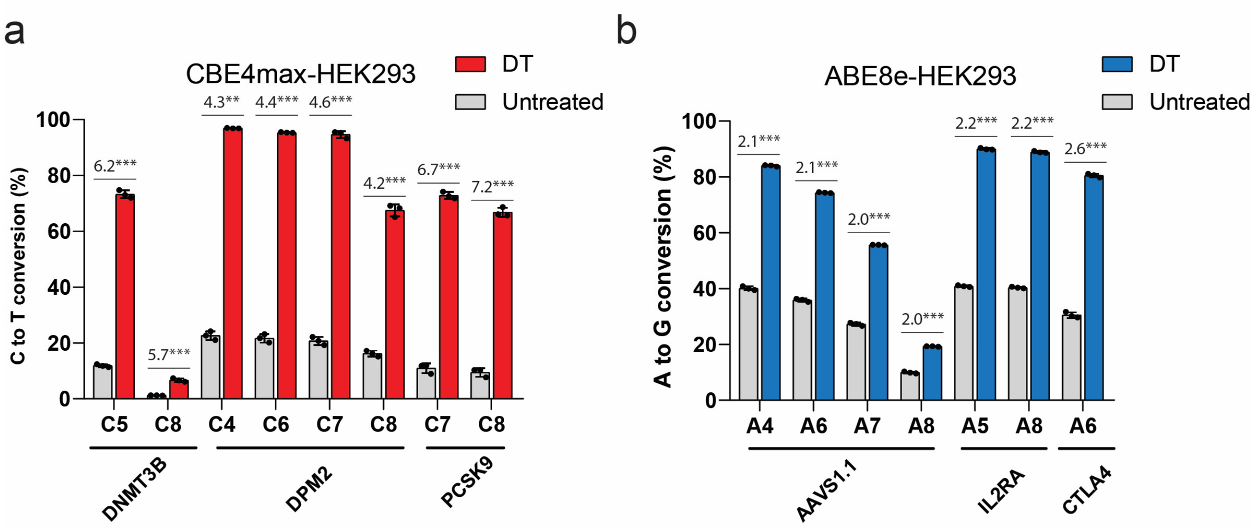

Supplementary Figure 3. **Co-selection increases efficiency of CBE4max and ABE8e.** (a) Bar graph of co-selected cytidine base editing events at indicated loci in HEK293 with CBE4max, with or without DT selection, showing C-T conversion (%). (b) Bar graph of co-selected adenosine base editing events in HEK293 with ABE8e, with or without DT selection, showing A-G conversion (%). In all graphs, the values and error bars reflect mean ± s.d. of n=3 independent biological replicates. Relative fold-changes between DT-selected and non-selected cells are indicated in the graphs. *P< 0.05, **P< 0.01, ***P<0.001, Student's paired t-test (two-tailed). P values are calculated as below: in panel (a), *DNMT3B* (C5=0.00026, C8=0.00475), *DPM2* (C4=0.00017, C6=0.00016, C7=0.00004, C8=0.00017) *PSCK9* (C7=0.00033, C8=0.00020); in panel (b), *AAVS1.1* (A4=0.00004, A6=0.00007, A7=0.00018, A8=0.00077), *IL2RA* (A5=0.00003, A8=0.00002), *CTLA4* (A6=0.00004).

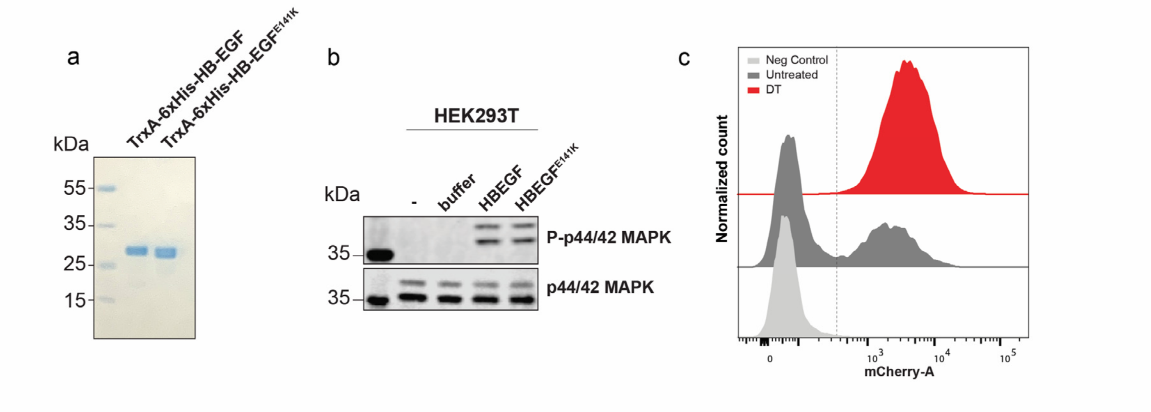

Supplementary Figure 4. **Expression of mCherry in the Xential knock-in cells after DT selection.** (a) SDS-PAGE gel stained with coomasie stain presenting the purified soluble HBEGF or $HBEGF^{E141K}$. Source data of Supplementary Figure 4a are provided as a Source Data file. (b) Western blot analysis of p44/42 MAPK and Phospho-p44/42 MAPK in cells treated with wildtype HBEGF and $HBEGF^{E141K}$. Phosphorylation of p44/42 MAPK represents one major downstream signalling of EGFR activation[1]. Representative results were shown from two independent biological replicates. Source data of Supplementary Figure 4b are provided as a Source Data file. (c) HEK293 cells were co-transfected with SpCas9, sgRNAIn3 and pHMEJ targeting *HBEGF* locus and then cultivated with or without DT selection. Cells were analyzed by flow cytometry for mCherry expression. Neg Control represent cells transfected with the control sgRNA instead of the sgRNAIn3. Representative histogram was shown for each condition with normalized cell count as the y-axis and mCherry (knock-in at *HBEGF* locus) as x-axis.

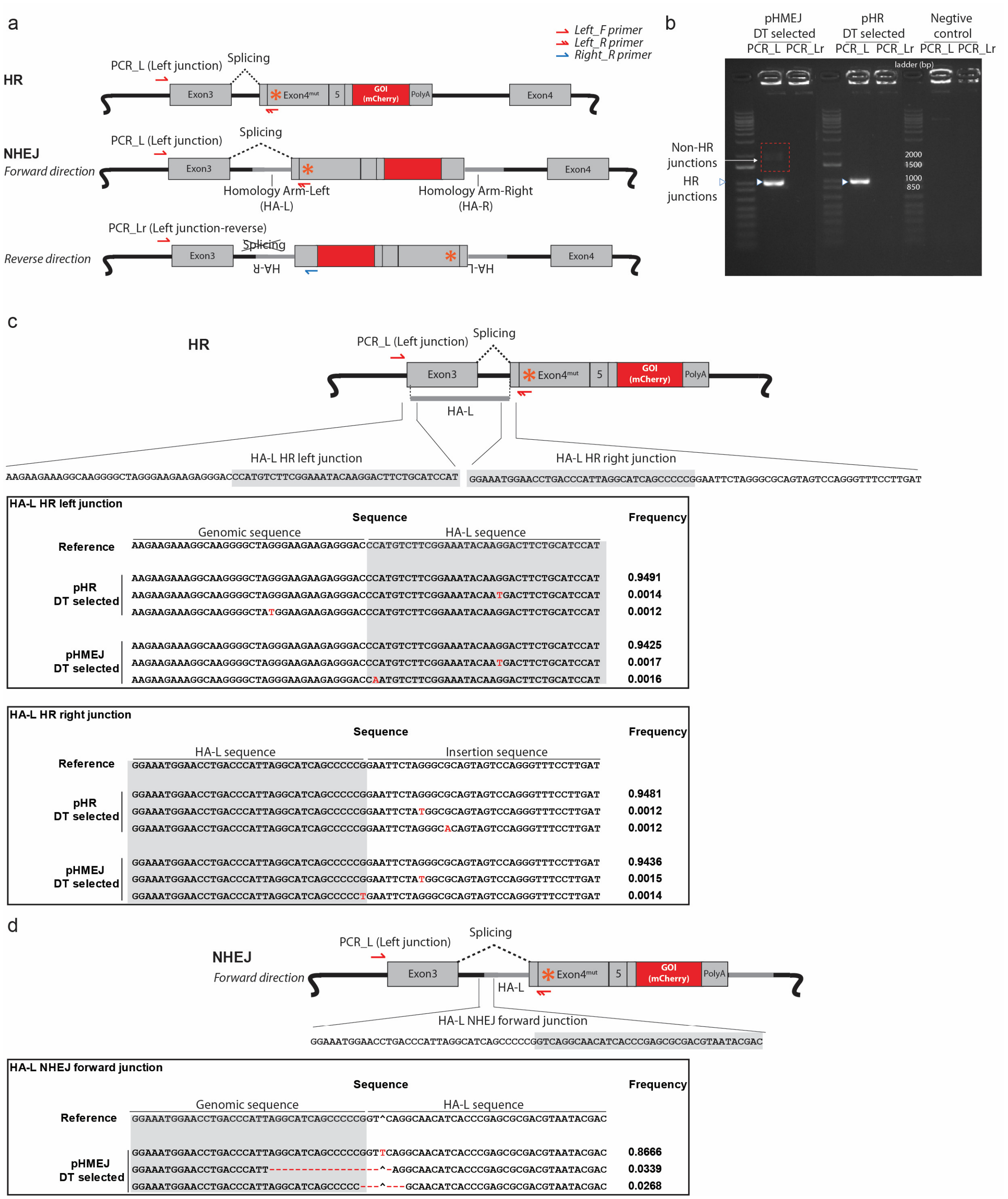

Supplementary Figure 5. Analysis of the DNA sequence at the junction between inserted DNA and genomic DNA at the *HBEGF* locus. (a) PCR primers were designed to amplify all types of left junctions of the insertion formed by the HR or NHEJ pathways. DT-selected samples with the pHMEJ- or pHR-mediated Xential insertions were analyzed; wild-type cells served as a negative control. (b) DNA electrophoresis on agarose gel pictures presenting genotyping results. PCR products matching the size of

HR junctions are marked by an arrow. PCR products of different sizes are indicated by a red rectangle. Representative results were shown from three independent biological replicates. (c) Amplicon-seq results presenting DNA sequences around the left HR junctions after the pHR or pHMEJ insertions. The top three variants are shown with their corresponding frequencies. Insertions, deletions or substitutions are highlighted in red. (c) Amplicon-seq results presenting DNA sequences around the left NHEJ junctions were amplified and analyzed by NGS. Top three variants are shown with their corresponding frequencies. Insertions, deletions or substitutions are highlighted in red.

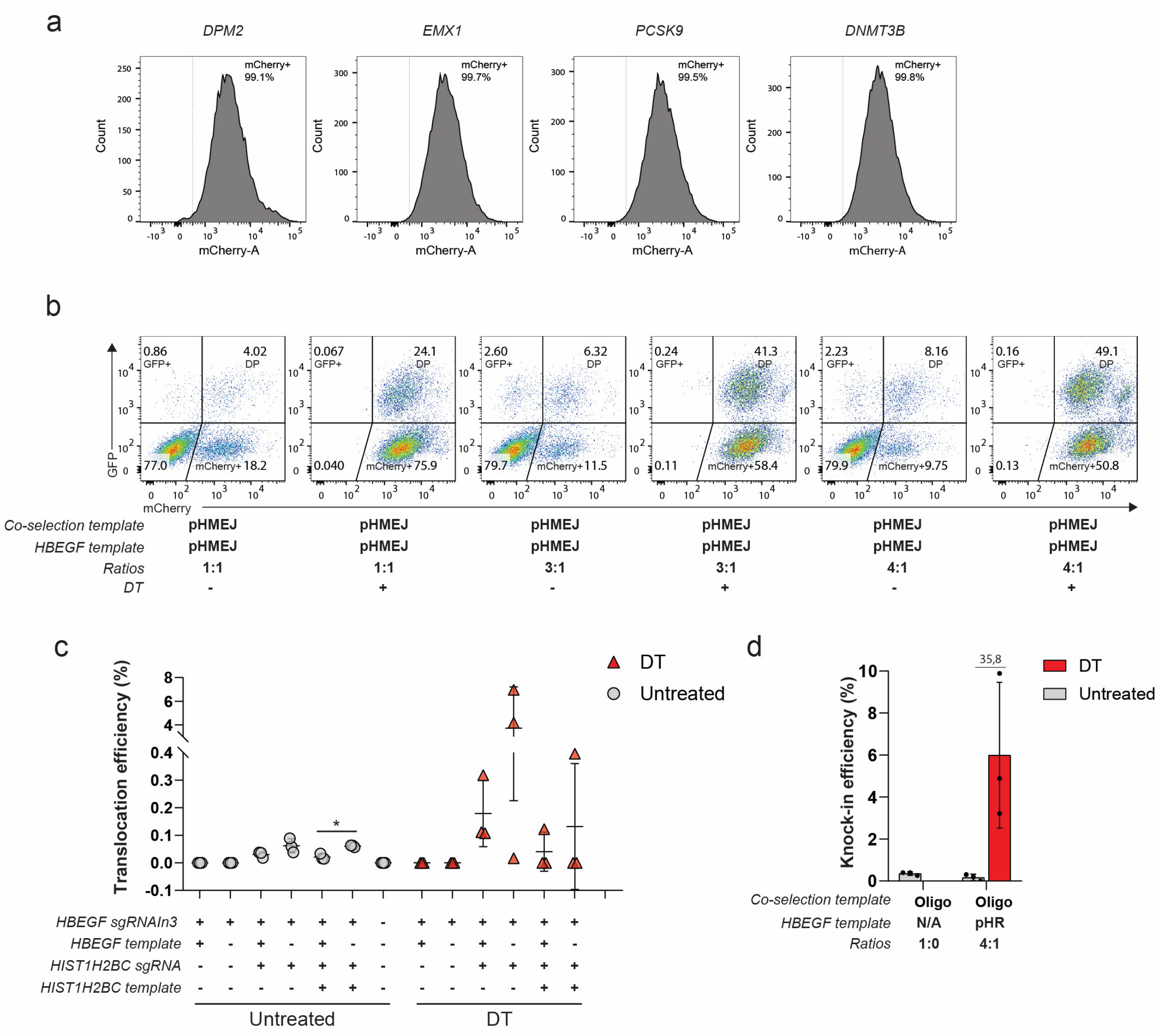

Supplementary Figure 6. **Xential co-selection of knock-out and knock-in events.** (a) Xential surviving populations co-selected for knock-out events maintained mCherry expression. Each target sgRNA was co-transfected with SpCas9, sgRNAIn3 and pHMEJ targeting *HBEGF* locus into HEK293 cells. DT selected cells then were analyzed by flow cytometry. Representative histogram shown for each condition with cell count as the y-axis and mCherry (knock-in at *HBEGF* locus) as x-axis. (b) Xential surviving populations co-selected for knock-in events maintained mCherry expression. pHMEJ and sgRNA targeting *HIST1H2BC* locus were co-transfected with SpCas9, sgRNAIn3 and pHMEJ targeting *HBEGF* locus into HEK293 cells at different weight ratios. DT selected and unselected cells were analyzed by flow cytometry. Representative scatter plot shown for each condition with GFP (knock-in at *HIST1H2BC* locus) as the y-axis and mCherry (knock-in at *HBEGF* locus) as x-axis. DP, double positives. (c) Analysis of translocation

events in multiplex genome editing experiment. The graph presenting the translocation frequency based on the BioRad ddPCR Copy Number Assay. ddPCR primers and probes were designed to detect the balanced translocation between *HBEGF* and *HIST1H2BC*. AP3B1 was used as the reference assay for calculating frequencies. (d) Xential co-selection of oligo knock-in events. Oligo template and sgRNA targeting *CD34* locus was transfected or co-transfected with SpCas9, sgRNAIn3 and pHMEJ targeting *HBEGF* locus into HEK293 cells, respectively. Genomic DNA was extracted from DT selected and unselected cells and analyzed by Amplicon-Seq. Values and error bars reflect mean ± s.d. of n=3 independent biological replicates. *P< 0.05, **P< 0.01, ***P<0.001, Student's paired t-test (two-tailed). P values are calculated as below: in panel (c), P=0.0159; in panel (d), P=0.0960.

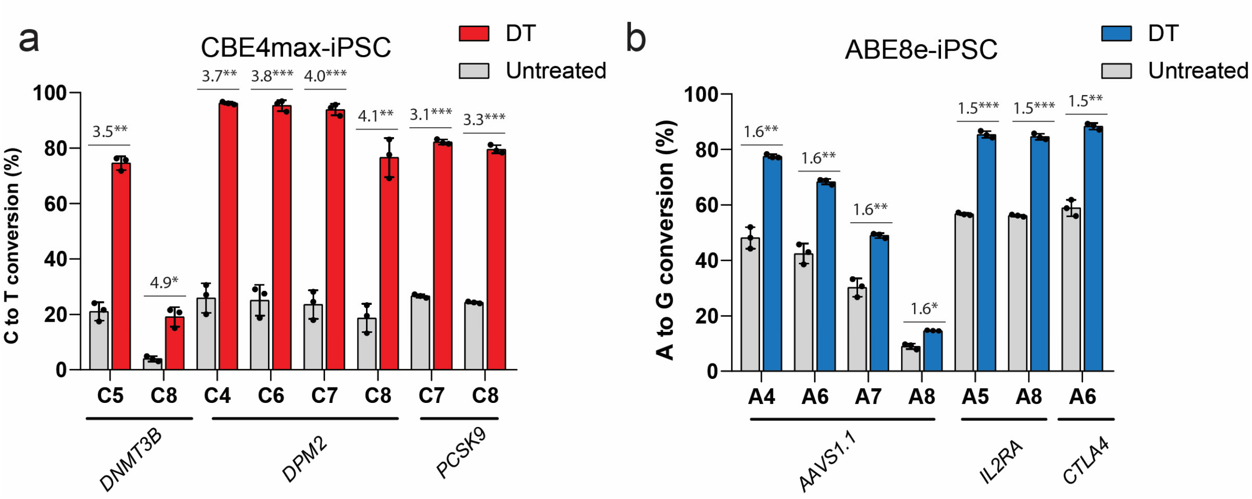

Supplementary Figure 7. Co-selection increases efficiency of CBE4max and ABE8e in hiPSC. (a) Bar graph of co-selected cytidine base editing events at indicated loci in hiPSC with CBE4max, with or without DT selection, showing C-T conversion (%). (b) Bar graph of co-selected adenosine base editing events in hiPSC with ABE8e,with or without DT selection, showing A-G conversion (%). In all graphs, the values and error bars reflect mean ± s.d. of n=3 independent biological replicates. Relative fold-changes between DT-selected and non-selected cells are indicated in the graphs. *P< 0.05, **P< 0.01, ***P<0.001, Student's paired t-test (two-tailed). P values are calculated as below: in panel (a), *DNMT3B* (C5=0.00359, C8=0.01749), *DPM2* (C4=0.00174, C6=0.00096, C7=0.00092, C8=0.00249) *PSCK9* (C7=0.00001, C8=0.00017); in panel (b), *AAVS1.1* (A4=0.00397, A6=0.00373, A7=0.00614, A8=0.001208), *IL2RA* (A5=0.00030, A8=0.00034), *CTLA4* (A6=0.00135).

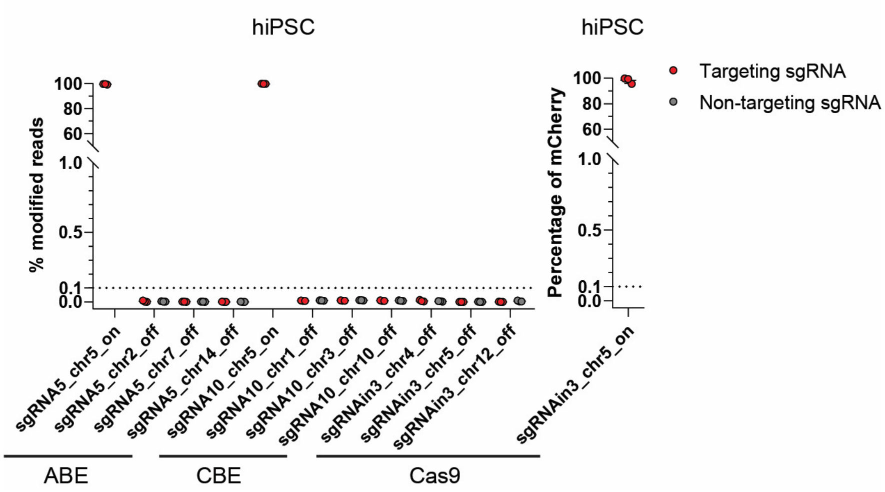

Supplementary Figure 8. The off-target analysis of sgRNA5, sgRNA10 and sgRNAin3. Data presenting efficiency of ABE, CBE and Cas9 at depicted loci analyzed by NGS. For sgRNAin3, the percentage of on-target modified reads was quantified as the percentage of mCherry knock-in cells. Non-targeting sgRNA was designed to contain no target sites in human genome and was used as negative control in this experiment. Values and error bars reflect mean ± s.d. of n=3 independent biological replicates.

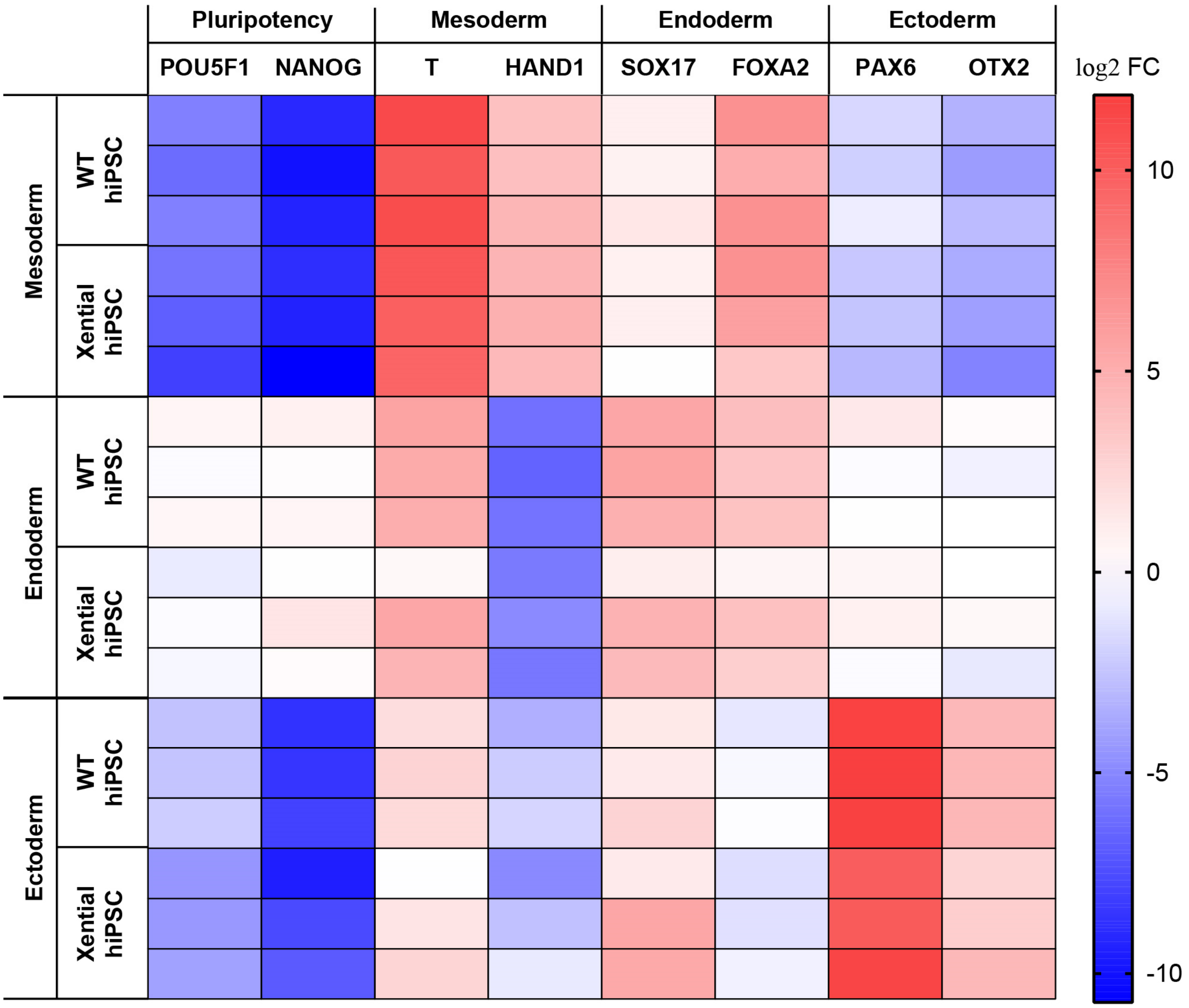

Supplementary Figure 9. The HBEGF^{E141K} mutation installed in hiPSC does not perturb differentiation process. Heatmap shows the qPCR data of the expression of two lineage-specific genes assayed upon differentiation to mesoderm, endoderm and ectoderm (fold change, log2 scale). The Xential hiPSCs were generated with Cas9/sgRNAin3/pHMEJ (Xential). The wild-type hiPSC (WT hiPSC) served as a positive control. Values were normalized to the expression of GAPDH and ACTB and set relative to undifferentiated controls (0) (data not included in the graph). Each differentiation condition was performed in triplicates (n=3) with n=2 qPCR technical repeats.

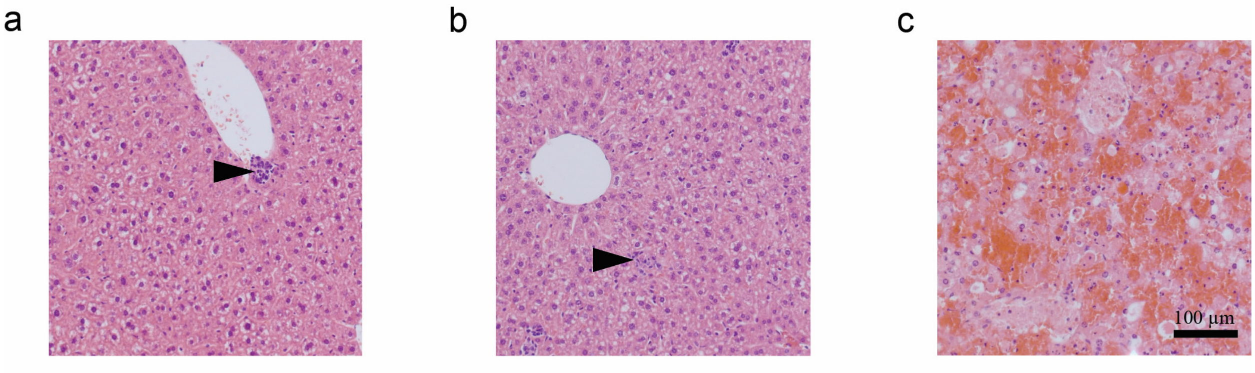

Supplementary Figure 10. **Establishing DT dose to generate mild liver damage.** Representative images of severity of liver damage (a). Untreated mouse, small foci of inflammatory cells (arrowhead) (b) 200 ng/kg DT, minimal liver damage, small foci of inflammatory cells (arrowhead) (c) 400 ng/kg DT, severe injury, hepatocellular coagulative necrosis and extravasation of erythrocytes. Increasing dose of DT dose results in greater liver toxicity. Representative photomicrographs from mice dosed with 0 ng/kg (n=2), 200 ng/kg (n=2) and 400 ng/kg (n=2).

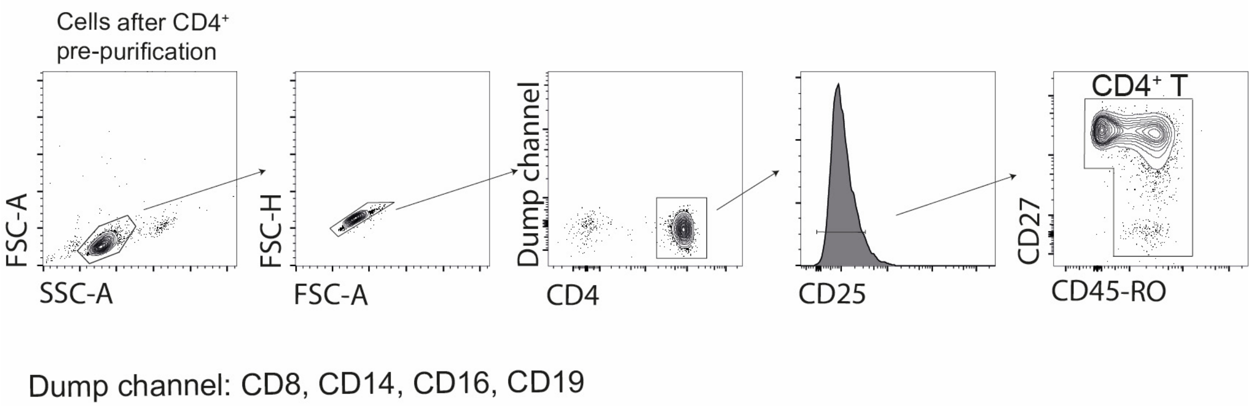

Supplementary Figure 11. Gating strategy for purification of CD4+ T cells. Total CD4+T cells were gated on live lymphocytes (FSC/SSC) and single cell fractions (FSC-A/FSC-H/FSC-W). The cells were then sorted based on the following gating strategy: CD4+, CD45RO+/-, CD27+/-, CD8-, CD14-, CD16-, CD19-, CD25- cell surface markers.

Supplementary Table 1. Off-target analysis of sgRNA5, sgRNA10 and sgRNAin3

Table presenting top 3 off-target sites selected for analysis. Mismatches or DNA/RNA bulges are highlighted in red.

	Name	Target sequence	Off-target sequence	chromosome
ABE	sgRNA5_chr2_off	GCAAATATGTGAAGGAGCTCNGG	GCAAATATGTGAAaGA-CTCTGG	chr2
	sgRNA5_chr7_off	GCAAATATGTGAAGGAGCTCNGG	GaAAATAT-TGAAGGAGCTCTGG	chr7
	sgRNA5_chr14_off	GCAAATATGTGAAGGAGCTCNGG	GCAAATAaGTGAAGGAGC-CAGG	chr14
CBE	sgRNA10_chr1_off	CAC-CTCTCTCCATGGTAACCNGG	CAC^T^CTCTCTCCAgGGTAACCAGG	chr1
	sgRNA10_chr3_off	CACCTCTCTCCATGGTAACCNGG	CA-CTCTCTgCATGGTAACCAGG	chr3
	sgRNA10_chr10_off	CACCTCTCTCCATGGTAACCNGG	CA-CTCTCTCCcTGGTAACCAGG	chr10
Cas9	sgRNAin3_chr4_off	GGGTGATGTTGCCTGACCGGNGG	G-cTGATGTTGCCTaACCGGGGG	chr4
	sgRNAin3_chr5_off	GGGTGATGTTGCCTGACCGGNGG	GGGTGAT-TTGCCTGAatGGAGG	chr5
	sgRNAin3_chr12_off	GGGTGATGTTGCCTGACCGGNGG	GGGTGgTGTTGCCTG-CCtGTGG	chr12

Supplementary Table 2. Amount of plasmid DNA transfected in HEK293, HCT116 and PC9-BFP experiments

**Transfection**					
	Genome editor/ sgRNA	Genome editor/ sgRNA1/ sgRNA2	Genome editor/ sgRNA1/ *HBEGF* repair template	Genome editor/ sgRNA1/ *HBEGF* repair template/ sgRNA2	Genome editor/ sgRNA2/ target repair template
Genome editor (SpCas9/CBE3/ABE7.10)	400 ng	400 ng	160 ng	160 ng	160 ng
sgRNA1 (Selection sgRNA)	100 ng	50 ng	40 ng	20 ng	
sgRNA2 (Target sgRNA)		50 ng		20 ng	40 ng
HBEGF repair template			400 ng	400 ng	
Target repair template					400 ng

Supplementary Table 3. Amount of transfected plasmid DNA in Xential mediated co-selection experiments

**Transfection**					
	Target pHR:	Target pHMEJ:	Target pHMEJ:	Target pHMEJ:	Target oligos:
	HBEGF pHR	*HBEGF* pHMEJ	*HBEGF* pHMEJ	*HBEGF* pHMEJ	*HBEGF* pHR
	2:1	1:1	3:1	4:1	2:1
Genome editor (SpCas9/CBE3/ABE7.10)	160 ng	160 ng	160 ng	160 ng	160 ng
sgRNA1 (Selection sgRNA)	13.3 ng	20 ng	10 ng	8 ng	13.3 ng
sgRNA2 (Target sgRNA)	26.7 ng	20 ng	30 ng	32 ng	26.7 ng
HBEGF repair template	133 ng	200 ng	100 ng	80 ng	133 ng
Target repair template	267 ng	200 ng	300 ng	320 ng	
Target oligo					267 ng

Supplementary Table 4. Amount of transfected plasmid DNA in iPSCs experiments

**Transfection**			
	Genome editor/ sgRNA	Genome editor/ sgRNA1/ sgRNA2	Genome editor/ sgRNA1/ *HBEGF* repair template
Genome editor (SpCas9/CBE3/ABE7.10)	200 ng	200 ng	66 ng
sgRNA1 (Selection sgRNA)	50 ng	25 ng	17 ng
sgRNA2 (Target sgRNA)		25 ng	
HBEGF repair template			167 ng

Supplementary Table 5. Amount of transfected plasmid DNA in translocation experiments

**Transfection**							
	1	2	3	4	5	6	7
SpCas9	160 ng	160 ng	160 ng	160 ng	160 ng	160 ng	160 ng
sgRNAin3	40 ng	40 ng	40 ng	40 ng	40 ng	40 ng	
sgRNA_*HIST1H2BC*		40 ng	40 ng		40 ng	40 ng	
HBEGF repair template (pHMEJ)	200 ng	200 ng	200 ng				
HIST1H2BC repair template (pHMEJ)			200 ng			200 ng	
Neg Control_sgRNA	240 ng	200 ng		440 ng	400 ng	200 ng	480 ng
**24 well format**							

Supplementary Table 6. Primers, probes and thermocycling conditions for ddPCR

**Information for ddPCR**					
**Sequence of ddPCR primers and probe for *HBEGF-HIST1H2BC* translocation detection**					
		**Forward sequence:** CTGGCCACAGCTCTTTTAGT			
		**Reverse sequence:** AGGAGAGAGGCTAAGGAACC			
		**Probe Sequence:** TGGGTAGGTGTGTGGGTGGAGGGGC			
		**Fluorophore:** FAM			
**Conditions for thermocycling**					
		Cycling step	Temperature	Time	Ramp rate
	1	Enzyme acivation	95	10 min	2.0° C/sec
	2	Denaturation	94	30 sec	2.0° C/sec
	3	Annealing and extension	61	1 min	
	4	Go to step 2 39x			
	5	Enzyme deactivation	98	10 min	2.0° C/sec
	6	Hold	4	Infinite	2.0° C/sec

Supplementary Reference

1. Oda, K., Matsuoka, Y., Funahashi, A. & Kitano, H. A comprehensive pathway map of epidermal growth factor receptor signaling. *Mol. Syst. Biol.* **1**, 2005.0010 (2005).
