# Supplementary material for: Universal toxin-based selection for precise genome engineering in human cells
Source: Nat Commun. 2021 Jan 21;12:497. doi: 10.1038/s41467-020-20810-z (PMC7820243; doi:10.1038/s41467-020-20810-z)
Supplement: Supplementary file 5 — Description of Additional Supplementary Files [file 41467_2020_20810_MOESM5_ESM.pdf]

Title: Supplementary Data 1.
Description: Sequences of primers used in this study

Title: Supplementary Data 2.
Description: DNA sequences of plasmids used in this study

Title: Supplementary Data 3.
Description: List of in silico predicted off-target sites for sgRNA10, sgRNA5 and sgRNAin3
