# Supplementary material for: Universal toxin-based selection for precise genome engineering in human cells
Source: Nat Commun. 2021 Jan 21;12:497. doi: 10.1038/s41467-020-20810-z (PMC7820243; doi:10.1038/s41467-020-20810-z)
Supplement: Supplementary file 6 — Reporting Summary [file 41467_2020_20810_MOESM6_ESM.pdf]

natureresearch

Corresponding author(s): Songyuan Li, Grzegorz Sienski, Marcello Maresca

Last updated by author(s): Dec 2, 2020

Reporting Summary

Nature Research wishes to improve the reproducibility of the work that we publish. This form provides structure for consistency and transparency in reporting. For further information on Nature Research policies, see Authors & Referees and the Editorial Policy Checklist .

Statistics

For all statistical analyses, confirm that the following items are present in the figure legend, table legend, main text, or Methods section.

n/a	Confirmed	
☐	☒	The exact sample size (*n*) for each experimental group/condition, given as a discrete number and unit of measurement
☐	☒	A statement on whether measurements were taken from distinct samples or whether the same sample was measured repeatedly
☐	☒	The statistical test(s) used AND whether they are one- or two-sided<br>*Only common tests should be described solely by name; describe more complex techniques in the Methods section.*
☒	☐	A description of all covariates tested
☒	☐	A description of any assumptions or corrections, such as tests of normality and adjustment for multiple comparisons
☐	☒	A full description of the statistical parameters including central tendency (e.g. means) or other basic estimates (e.g. regression coefficient) AND variation (e.g. standard deviation) or associated estimates of uncertainty (e.g. confidence intervals)
☐	☒	For null hypothesis testing, the test statistic (e.g. *F*, *t*, *r*) with confidence intervals, effect sizes, degrees of freedom and *P* value noted<br>*Give P values as exact values whenever suitable.*
☒	☐	For Bayesian analysis, information on the choice of priors and Markov chain Monte Carlo settings
☒	☐	For hierarchical and complex designs, identification of the appropriate level for tests and full reporting of outcomes
☒	☐	Estimates of effect sizes (e.g. Cohen's *d*, Pearson's *r*), indicating how they were calculated

Our web collection on statistics for biologists contains articles on many of the points above.

Software and code

Policy information about availability of computer code

Data collection	NGS sequencing data were demultiplexed using bcl2fastq software (version 2.20.0.422), and individual FASTQ files were analyzed using a Perl (version 5.26.1) implementation of the Matlab script described previously (see Komor et al Nature 2016).<br>Off-target analysis was peroformed using CRISPResso2.<br>Data analysis of ddPCR was performed using QuantaSoft (Bio-Rad).<br>Data analysis of qPCR was performed using GenEx v7.1 software (MultiD Analyses AB).
Data analysis	Frequency, mean, and standard deviations were calculated using GraphPad Prism 8. Flowjo_v10.6.1 was used for flow cytometry analysis.

For manuscripts utilizing custom algorithms or software that are central to the research but not yet described in published literature, software must be made available to editors/reviewers. We strongly encourage code deposition in a community repository (e.g. GitHub). See the Nature Research guidelines for submitting code & software for further information.

Data

Policy information about availability of data

All manuscripts must include a data availability statement. This statement should provide the following information, where applicable:

- Accession codes, unique identifiers, or web links for publicly available datasets
- A list of figures that have associated raw data
- A description of any restrictions on data availability

Raw NGS data available on request from the corresponding author.

Field-specific reporting

Please select the one below that is the best fit for your research. If you are not sure, read the appropriate sections before making your selection.

☒ Life sciences ☐ Behavioural & social sciences ☐ Ecological, evolutionary & environmental sciences

For a reference copy of the document with all sections, see nature.com/documents/nr-reporting-summary-flat.pdf

Life sciences study design

All studies must disclose on these points even when the disclosure is negative.

Sample size	Sample sizes were determined based on literature precedence for genome editing experiments (see Komor et al Nature 2016). For in vivo work, sample size calculations were performed based on possible experimental variability from DT treatment and sample sizes required for downstream molecular biology analysis of samples.
Data exclusions	No data was excluded.
Replication	All cell experiments were repeated at least once. All attempts at replication were successful. The animal experiments were performed once with six biological independent mice used in each group.
Randomization	For in vitro work, mammalian cells used in this study were grown under identical conditions; no randomization was used. For in vivo work, animals were randomized to groups based on their sex prior to start of the experiment.
Blinding	For in vitro work, mammalian cells used in this study were grown under identical conditions; blinding was not used. For in vivo work, investigators were blinded to group allocation during data collection and analysis.

Reporting for specific materials, systems and methods

We require information from authors about some types of materials, experimental systems and methods used in many studies. Here, indicate whether each material, system or method listed is relevant to your study. If you are not sure if a list item applies to your research, read the appropriate section before selecting a response.

Materials & experimental systems

n/a	Involved in the study	
☐	☒	Antibodies
☐	☒	Eukaryotic cell lines
☒	☐	Palaeontology
☐	☒	Animals and other organisms
☐	☒	Human research participants
☒	☐	Clinical data

Methods

n/a	Involved in the study	
☒	☐	ChIP-seq
☐	☒	Flow cytometry
☒	☐	MRI-based neuroimaging

Antibodies

Antibodies used	The following antibodies were purchased from BD Biosciences: CD4-PECF594 (RPA-T4: cat.no. 562281), CD25-PECy7 (M-A251; cat.no. 557741), CD8-APCCy7 (RPA-T8; cat.no. 557760), CD14-APCCy7 (MϕP-9; cat.no. 557831), CD16-APCCy7 (3G8; cat.no. 557758), CD19-APCCy7 (SJ25-C1; cat.no. 557791), CD45RO-BV510. (UCHL1; 563215). Fixable Viability Dye eFluor™ 780 from ThermoFisher (cat.no. 65-0865) was used to exclude dead cells.
Validation	All antibodies listed above were purchased commercially (e.g. https://www.bdbiosciences.com/ds/pm/tds/562316.pdf) and tested in the lab for their performance.

Eukaryotic cell lines

Policy information about cell lines

Cell line source(s)	HEK293 (ATCC), HCT116 (ATCC), PC9 (ATCC).
Authentication	Cells were authenticated by the supplier using STR analysis.
Mycoplasma contamination	All cell lines tested negative for mycoplasma
Commonly misidentified lines (See ICLAC register)	None used.

Animals and other organisms

Policy information about studies involving animals; ARRIVE guidelines recommended for reporting animal research

Laboratory animals	Experimental mice were generated as double heterozygotes by breeding Alb-Cre mice (016833, The Jackson Laboratory) to iDTR mice (Expression of transgene, human HBEGF, is blocked by loxP-flanked STOP sequence) on the C57BL/6NCrl genetic background. The experiment was performed using 6-month-old mice, 6 male and 6 female, randomized into 2 groups with equal male and female mice in each group. The manuscript contains details covering all of the Essential 10 of the ARRIVE guidelines.
Wild animals	The study did not involve wild animals.
Field-collected samples	The study did not involve samples collected from the field.
Ethics oversight	All mouse experiments were approved by the AstraZeneca internal committee for animal studies and the Gothenburg Ethics Committee for Experimental Animals (license number: 162–2015+) compliant with EU directives on the protection of animals used for scientific purposes.

Note that full information on the approval of the study protocol must also be provided in the manuscript.

Human research participants

Policy information about studies involving human research participants

Population characteristics	Healthy donors were recruited from AstraZeneca volunteers and all samples were taken following appropriate blood collection guidelines. All blood donor volunteers signed Informed Consent form and donation was approved by AstraZeneca's Institutional review board and local Ethic committee (033-10).
Recruitment	Healthy donors were recruited from AstraZeneca volunteers and all samples were taken following appropriate blood collection guidelines. All blood donor volunteers signed Informed Consent form and donation was approved by AstraZeneca's Institutional review board and local Ethic committee (033-10).
Ethics oversight	All blood donor volunteers signed Informed Consent form and donation was approved by AstraZeneca's Institutional review board and local Ethic committee (033-10).

Note that full information on the approval of the study protocol must also be provided in the manuscript.

Flow Cytometry

Plots

Confirm that:

- [x] The axis labels state the marker and fluorochrome used (e.g. CD4-FITC).
- [x] The axis scales are clearly visible. Include numbers along axes only for bottom left plot of group (a 'group' is an analysis of identical markers).
- [ ] All plots are contour plots with outliers or pseudocolor plots.
- [x] A numerical value for number of cells or percentage (with statistics) is provided.

Methodology

Sample preparation	Total CD4+ T cells isolation: Peripheral blood mononuclear cells were isolated from fresh blood using Lymphoprep (STEMCELL Technologies) density gradient centrifugation and total CD4+ T cells were enriched by negative selection with the EasySep Human CD4+ T Cell Enrichment Kit (STEMCELL Technologies). Enriched CD4+ T cells were then futher purified by fluorescence-activated cell sorting (FACSAria III, BD Biosciences) based on exclusion of CD8+ CD14+ CD16+ CD19+ CD25+ cell surface markers to an average purity of 98%.
Instrument	Cell sorting was performed using BD FacsARIA III using a 70µm nozzles. Cell analysis was performed using BD Fortessa (BD Biosciences).
Software	Acquisition was done using the FACSDiva software (BD Biosciences) and final analysis was performed using the FlowJo software (TreeStar).
Cell population abundance	Sorted total CD4+ T cells were isolated from three different donors. They were purified by fluorescence-activated cell sorting (FACSAria III, BD Biosciences) based on exclusion of CD8+, CD14+, CD16+, CD19+, CD25+ cell surface markers to an average purity of 98%.

Gating strategy

Total CD4+T cells were gated on live lymphocytes (FSC/SSC) and single cell fractions (FSC-A/FSC-H/FSC-W). The cells were then sorted based on the following gating strategy: CD4+, CD45RO+/-, CD27+/-, CD8-, CD14-, CD16-, CD19-, CD25- cell surface markers.

☒ Tick this box to confirm that a figure exemplifying the gating strategy is provided in the Supplementary Information.
